# Genetic Diversity of African Trypanosomes in Tsetse Flies and Cattle From the Kafue Ecosystem

**DOI:** 10.3389/fvets.2021.599815

**Published:** 2021-01-27

**Authors:** Yukiko Nakamura, Kyoko Hayashida, Victoire Delesalle, Yongjin Qiu, Ryosuke Omori, Martin Simuunza, Chihiro Sugimoto, Boniface Namangala, Junya Yamagishi

**Affiliations:** ^1^Graduate School of Infectious Diseases, Hokkaido University, Sapporo, Japan; ^2^Research Center for Zoonosis Control, Hokkaido University, Sapporo, Japan; ^3^International Collaboration Unit, Research Center for Zoonosis Control, Hokkaido University, Sapporo, Japan; ^4^Melindika, Non-governmental Organization of International Solidarity, Itezhi-Tezhi, Zambia; ^5^Department of Disease Control, School of Veterinary Medicine, The University of Zambia, Lusaka, Zambia; ^6^Department of Para-Clinical Studies, School of Veterinary Medicine, The University of Zambia, Lusaka, Zambia

**Keywords:** *Trypanosoma vivax*, *Trypanosoma vivax* bovine trypanosomosis, *Trypanosoma vivax*-like, African animal trypanosomosis, cathepsin L-like cysteine protease, anemia

## Abstract

We clarified the genetic diversity of *Trypanosoma* spp. within the Kafue ecosystem, using PCR targeting the internal transcribed spacer 1 and the cathepsin L-like cysteine protease (CatL) sequences. The overall prevalence of *Trypanosoma* spp. in cattle and tsetse flies was 12.65 and 26.85%, respectively. Cattle positive for *Trypanosoma vivax* had a significantly lower packed cell volume, suggesting that *T. vivax* is the dominant *Trypanosoma* spp. causing anemia in this area. Among the 12 operational taxonomic units (OTUs) of *T. vivax* CatL sequences detected, one was from a known *T. vivax* lineage, two OTUs were from known *T. vivax*-like lineages, and nine OTUs were considered novel *T. vivax*-like lineages. These findings support previous reports that indicated the extensive diversity of *T. vivax*-like lineages. The findings also indicate that combining CatL PCR with next generation sequencing is useful in assessing *Trypanosoma* spp. diversity, especially for *T. vivax* and *T. vivax*-like lineages. In addition, the 5.42% prevalence of *Trypanosoma brucei rhodesiense* found in cattle raises concern in the community and requires careful monitoring of human African trypanosomiasis.

## Introduction

Diseases caused by the infection of Salivaria *Trypanosoma* spp. are of medical and veterinary importance in sub-Saharan Africa. Human African trypanosomiasis (HAT) is caused by *Trypanosoma brucei rhodesiense* and *T. b. gambiense* in East and West Africa, respectively. African animal trypanosomosis (AAT) is caused by various *Trypanosoma* spp. from the subgenera *Duttonella* (*Trypanosoma vivax*), *Nannomonas* (*Trypanosoma congolense, Trypanosoma simiae*), and *Trypanozoon* (*T. b. brucei*). Salivaria *Trypanosoma* spp. are transmitted solely by tsetse flies (*Glossina* spp.), except for *T. vivax*, in which mechanical transmission by biting flies has been described (1).

African animal trypanosomosis, especially bovine trypanosomosis, is of national importance in Zambia. The prevalence of infection is generally high as a result of the continuous infestation of tsetse flies, abundant wildlife reservoirs, and cattle rearing in tsetse infested areas. As a result, bovine trypanosomosis remains a significant constraint to rural development in large areas of western, southern, and eastern Zambia (2). Zambia currently reports fewer than 100 new HAT cases annually, mainly from areas within the Luangwa River valley in eastern Zambia (3). Several HAT cases have been reported from re-emerging HAT foci outside the major HAT foci, such as the case in Kafue National Park in 2016, which had been more than 50 years since the last documented case (4). This suggests that the human infective *T. b. rhodesiense* is being maintained within this ecosystem, which puts the local community at the risk of HAT.

Bovine trypanosomosis in Zambia is mainly caused by *T. congolense* and *T. vivax*, with some cases caused by *Trypanozoon* (*T. b. brucei*). Previous studies have detected a high prevalence of these *Trypanosoma* spp. in the eastern and southern province of Zambia (33.5% prevalence, 29.3% prevalence among anemic cattle, respectively), where the majority of infections were attributed to *T. congolense* (5, 6). The clinical signs of bovine trypanosomosis include anemia, relapsing fever, enlarged lymph nodes, reduced milk yield, reduced productivity, decreased fertility, and, in severe cases, abortion, emaciation, and eventual death (7). Since these symptoms are non-specific, diagnosis of bovine trypanosomosis requires the detection of the parasite, its antigen, or antibodies against the parasite. The most widely used technique is parasite detection in light microscopy observation of wet blood films or Giemsa-stained thick and thin fixed blood films. Increased sensitivity can be obtained when hematocrit centrifugation technique or the buffy coat technique is used to concentrate the parasite load (8). However, these techniques cannot differentiate the *Trypanosoma* spp. intraspecifically, and are not sensitive enough to detect low parasite levels (9). Antibody detection methods, such as indirect ELISA, have been widely used in epidemiological studies. However, these methods do not prove on-going infection and cannot differentiate infection between *Trypanosoma* spp. (9). Molecular methods, such as PCR and loop-mediated isothermal amplification, have been used to establish species-specific and cross-species detection, and can detect intraspecific diversity. An example is PCR targeting the internal transcribed spacer (ITS) region of ribosomal genes (10, 11). This method has been commonly used due to its high sensitivity attributed to the high copy number and the feasibility of the inter-species length variation enabling visual discrimination by gel electrophoresis. Sequencing the amplicon can further elevate the sensitivity and allow intraspecific identification of *T. congolense* and, to some extent, *T. vivax* (12). However, to elucidate the highly complex disease epidemiology and genetic diversity of *T. congolense* and *T. vivax*, molecular methods with the resolution to distinguish beyond the *T. congolense* subgroups or *T. vivax* lineages are needed.

*Trypanosoma congolense* comprises of three subgroups (savannah, forest, and kilifi) that vary in virulence, pathogenicity, and geographical distribution (13). The three subgroups coexist in Zambia (14). In experimental infections of susceptible zebu cattle (Bos indicus), kilifi was non-pathogenic, forest was poorly pathogenic, and savannah was the most virulent subgroup (13). The savannah subgroup is reportedly the most genetically divergent and widespread across sub-Saharan Africa. Examinations of savannah phenotypes revealed markedly differing virulence and drug resistance, even in the same location (15, 16). The findings suggested that *T. congolense* undergoes genetic recombination in nature. This view is supported by previous population genetic studies based on microsatellites and population genomic research using whole-genome analyses (17, 18). Especially high diversity of *T. congolense* savannah was observed in Zambia, which was due to genetic exchange between phylogenetically distinct *T. congolense* savannah parasites (19).

The pathogenicity of *T. vivax* is known to differ between East and West Africa. West African *T. vivax* isolates are believed to be more pathogenic than East African isolates (20). However, severe hemorrhagic outbreaks with high mortality levels have also been reported in East Africa (7), and the correlation between the different isolates and disease severity in cattle remains unclear. While genetic homogeneity of isolates from West Africa and South America has been reported, greater genetic diversity among isolates from East Africa has been observed (21–30). In addition, *T. vivax* isolates that are genetic and morphologically distinct from the reference isolate *T. vivax* Y486 (isolated from Nigerian cattle) have been reported in East Africa (21–24). Therefore, parasites within the subgenus *Duttonella* separated by relative genetic distance from *T. vivax* Y486 have been widely termed as a “*T. vivax*-like” lineage (21, 23, 25–27). The taxonomic position of these trypanosomes is still unclear. It is possible that *T. vivax*-like trypanosomes correspond to other species within *Duttonella*, such as *T. uniforme* and *T. vivax ellipsiprymni*. Notably, the phylogenetic inference of *T. vivax* and *T. vivax*-like isolates from Mozambique suggested that a *T. vivax*-like lineage, TvL-Gorongosa, should be elevated to the species status (27).

Several genetic markers have been utilized to assess the genetic diversity of *T. congolense* and *T. vivax* at the intraspecific level. Representative markers include the glyceraldehyde 3-phosphate dehydrogenase gene (gGAPDH) sequence (31), fluorescent fragment length barcoding (FFLB) using the 18S and 28S ribosomal RNA regions (32), ITS rRNA sequences (ITS1, 5.8S, and ITS2) (24), and the cathepsin L-like cysteine protease (CatL) sequences (33), which all have their advantages and disadvantages. While gGAPDH sequences are valuable markers for identification of species and intraspecific diversity, the relationships within a subgroup of *T. congolense* or among the two *T. vivax* lineages (*T. vivax* and *T. vivax*-like) have not been well-resolved (27). Fluorescent fragment length barcoding displayed the highest sensitivity in detecting *Duttonella* trypanosomes from tsetse fly samples but could not distinguish within the two *T. vivax* lineages (32). ITS rRNA PCR and sequencing have high resolution and have been successful in illustrating the diversity within the *T. vivax* lineage and the *T. vivax*-like lineage. However, the sensitivity of this approach is inferior to FFLB and requires cloning and sequencing of several clones from each sample (27). CatL PCR displayed comparable sensitivity to FFLB (27), and has been used for genotyping a variety of *Trypanosoma* spp. (25, 33–36).

Kinetoplastid parasites express two C1 peptidases related to mammalian cathepsin B- and L (CatL)-like peptidases, which are either essential to survival or are important virulence factors contributing to disease pathogenesis (37). The pronounced genetic sequence diversity within the CatL sequences suggests that this region evolved early in evolution or has faster mutation rates to adapt to the parasites' divergent cellular functions (38). The high diversity in the CatL region between different *Trypanosoma* spp. has been proposed as a suitable genetic marker for analyzing the intraspecific diversity of *T. vivax* and *T. congolense*. Phylogenetic analysis of CatL sequences supported the phylogenetic relationship of trypanosomes based on the partial small subunit rRNA (V7-V8) gene sequence data (25). CatL genes have been successful in identifying nine clades of *T. vivax* (TviCatL1-9) throughout the West and East Africa and South America (25, 39), and five clades within the *T. congolense* savannah subgroup (SAV1-3, SAVna) (40). *Trypanosoma vivax* isolates from West Africa shared CatL clades with South America, including TviCatL1-4. *Trypanosoma vivax* CatL clades from East and Southern Africa showed divergent sequences, including TviCatL5–7 for isolates from Mozambique and TviCatL8–9 from Kenya (25). *Trypanosoma vivax* from Zambian cattle also formed one distinct clade and clustered with East African and Southern African sequences (39). Extending these studies to assess the genetic diversity of trypanosomes found in tsetse flies and cattle from the same ecosystem can enable a more in-depth understanding of the transmission of parasites and disease manifestation in cattle.

In this study, we aimed to elucidate the *Trypanosoma* spp. prevalence in tsetse flies and cattle from the Kafue ecosystem. Combined molecular methods were used for comprehensive detection and genotyping of African trypanosomes. ITS1 PCR was used to detect *Trypanosoma* spp. at the species level. CatL PCR coupled with next generation sequencing was used to illustrate the intraspecific diversity of *Trypanosoma* spp.

## Materials and Methods

### Study Area and Sample Collection

This study was performed in the Itezhi-Tezhi District, which is located in the south-western region of Central Province of Zambia, in the Kafue ecosystem. Kafue National Park is the oldest and largest national park in Zambia, covering an area of approximately 22,400 km^2^ (41). Kafue National Park is surrounded by several Game Management Areas (GMAs), which act as buffer zones for National Parks and mitigate the adverse effects of human activities. Licensed safari, subsistence hunting, and agricultural activities are permitted in the GMAs for local communities. The Nkala GMA covers an area of approximately 194 km^2^. There is no local population living in this area (42). Kafue National Park and the surrounding GMAs comprise the Kafue ecosystem, which is home to a wide variety of flora and fauna, including tsetse flies. *Glossina morsitans centralis* is the dominant tsetse fly species in this area, with a lower distribution of *Glossina pallidipes* (43). Therefore, in many communities adjacent to Nkala GMA, human settlement, cattle grazing areas, and tsetse infested areas overlap.

Cattle blood sampling was conducted in the five villages of Iyanda, New Ngoma, Ntubya, Kaminza, and Basanga in April and May of 2019 (Supplementary Figure 1). The major cattle breeds were crosses between local breeds (Tonga and Baila) and exotic breeds (mostly Boran and Brahman). The estimated cattle population within the community was 15,000 heads. Using Cochran's formula (44) with a 95% confidence level and a confidence interval of 5, the required sample size was computed to be 375 heads. Blood samples were collected from the jugular vein of the cattle using 18G needles and 5 mL syringes. Each sample was transferred to a heparin-lithium tube. A total of 498 blood samples were randomly collected from 65 farmers. The samples were immediately subjected to micro-hematocrit centrifugation to obtain packed cell volume (PCV) values (12,000 rpm, 5 min). Thin blood smears were also made, and Giemsa stained for microscopic observation of parasites. The remaining blood samples were preserved at 4°C until DNA extraction. Four epsilon traps were set for each sampling point to catch tsetse files. The traps were set once for 3 constructive days. The traps were visited each morning and afternoon to collect the captured flies. In areas with an insufficient number of catches, a mobile trap was used to supply the number of tsetse flies. Mobile trapping was conducted within a one-kilometer radius, which is within the average lifetime dispersal of morsitans group of tsetse flies (45). A total of 298 tsetse flies were captured from 10 sampling points (Supplementary Figure 2). The captured flies were inspected using a stereomicroscope for morphological identification of species and sex. The flies were stored in 2 mL sample tubes with silica beads to dry.

### DNA Extraction

DNA extraction of cattle blood samples was conducted using QuickGene DNA whole blood kit S (Kurabo, Osaka, Japan), following the manufacturer's protocol. The dried tsetse flies were transferred to new tubes with beads and smashed using a Micro Smash MS−100 bead cell disrupter (Tomy, Tokyo, Japan) at 3,000 rpm for 45 s. DNA was extracted using a modified protocol with the DNA Isolation Kit for Mammalian Blood (Roche, Basel, Switzerland). Briefly, 330 μL of white cell lysis buffer was added directly into each tube, vortexed, and heated at 37°C for 30 min. Then, 170 μL of protein precipitation solution was added, vortexed thoroughly, and centrifuged at 15,000 rpm for 20 min. DNA was precipitated by the addition of ethanol. All extracted DNA was stored at −30°C until further use.

### Tsetse Fly Species Confirmation Using *Glossina* Its PCR

For molecular confirmation of the tsetse fly species, GlossinaITS1_for (5′-GTG ATC CAC CGC TTA GAG TGA−3′) and GlossinaITS1_rev (5′-GCA AAA GTT GAC CGA ACT TGA−3′) primers were used to amplify the ITS1 region of ribosomal genes of tsetse flies (46). Reactions contained 1–10 ng of template DNA, 1 × Ampdirect Plus (Shimadzu Corp., Kyoto, Japan), 0.25 U BioTaq HS DNA Polymerase (Bioline, Memphis, TN, USA), 0.2 mM primers, and distilled water to a total volume of 10 μL. Amplification included an initial denaturation step at 95°C for 10 min, followed by 30 cycles each of 94°C for 30 s, 62°C for 1 min, 72°C for 2 min, and a final extension step at 72°C for 7 min. The band patterns were visually inspected after gel electrophoresis (*G. pallidipes*: 920 bp, *G. m. centralis*: 800 bp and 150 bp). There was a total of 212 *G. pallidipes* and 86 *G. m. centralis* samples.

### Human Infective *T. b. rhodesiense* Detection Using Serum Resistance-Associated (SRA) PCR

SRA284F (5′-ATA GTG ACA AGA TGC GTA CTC AAC GC−3′) and SRA284R (5′-AAT GTG TTC GAG TAC TTC GGT CAC GCT−3′) primers was used to detect human infective *T. b. rhodesiense* (47). The PCR reagents were the same as those described above for *Glossina* ITS PCR. Amplification included an initial denaturation step at 95°C for 10 min, followed by 40 cycles each of 94°C for 30 s, 60°C for 1 min, 72°C for 1 min, and a final extension step at 72°C for 2 min.

### ITS1 PCR for *Trypanosoma* spp. Detection and Species Identification

AITSF (5′-CGG AAG TTC ACC GAT ATT GC−3′) and AITSR (5′-AGG AAG CCA AGT CAT CCA TC−3′) primers were used to amplify the ITS1 region to analyze *Trypanosoma* spp. prevalence (12). The PCR reagents were the same as those of the *Glossina* ITS PCR described above. Amplification included an initial denaturation step at 95°C for 10 min, followed by 37 cycles of 94°C for 30 s, annealing at 58°C for 90 s, 72°C for 2 min, and a final extension step at 72°C for 7 min. *Trypanosoma* spp. were identified based on observation of gel electrophoresis.

### CatL PCR and Sequencing on the Illumina MiSeq Platform

PCR amplification of the CatL region was carried out for the ITS1 positive samples using DTO154/DTO155 primers (33), customized by attaching adapter sequences (Illumina, San Diego, CA, USA) to the 5′ ends DTO154illumina (5′-ACA CTC TTT CCC TAC ACG ACG CTC TTC CGA TCT NNA CAG AAT TCC AGG GCC AAT GCG GCT CGT GCT GG−3′), DTO155illumina(5′-GTG ACT GGA GTT CAG ACG TGT GCT CTT CCG ATC TNN TTA AAG CTT CCA CGA GTT CTT GAT GAT CCA GTA−3′). The reactions included 1–10 ng of genomic DNA, 0.2 mM primers, reagents from the KAPA Taq EXtra PCR Kit (Kapa Biosystems, Wilmington, MA, USA), using final concentrations of 1 × KAPA Taq EXtra buffer, 0.5 U KAPA Taq EXtra, 0.2 mM KAPA dNTP Mix, 1.5 mM MgCl2, and distilled water to a total volume of 10 μL. PCR grade water was used in place of genomic DNA as a negative control. Amplification included an initial denaturation step at 95°C for 10 min, followed by 15 cycles each of 94°C for 30 s, 60°C for 1 min, 72°C for 30 s, and a final extension step at 72°C for 10 min. The final PCR was done to attach a unique index to each sample to enable multiplexed sequencing using the Illumina platform. Reactions contained 1 mM Illumina dual-index primer mix, 1 × KAPA Taq EXtra buffer, 1 U KAPA Taq EXtra, 0.2 mM KAPA dNTP Mix, 1.5 mM MgCl2 and distilled water to a total volume of 20 μL. The PCR products were pooled in equal amounts into one library and analyzed using 2% agarose gel electrophoresis. The band of interest was cut and purified using the Wizard SV Gel and PCR Clean-Up System (Promega, Madison, WI, USA). Quantification of the library was done using a Qubit dsDNA HS assay kit and a Qubit fluorometer (Thermo Fisher Scientific, Waltham, MA, USA), and adjusted to 4 nM using nuclease-free water as the final library. The library was then applied to the Illumina MiSeq platform (Illumina MiSeq System, RRID:SCR_016379). MiSeq Reagent Kit v3 (Illumina) was used for 300 base pairs, paired-end sequencing. PhiX DNA spike-in control (25%) was added to increase the diversity of the amplicon library.

### Analysis and Assignment of CatL OTUs

The raw reads generated from CatL amplicon sequencing were polished, clustered at 95% identity, and the representative sequence was generated using the Amplicon Tool Kit (AMPtk) pipeline and default parameters of “amptk illumina,” “amptk dada2,” “amptk filter,” and “amptk lulu” (48). The representative sequence for each cluster was termed as an operational taxonomic unit (OTU). The generated CatL OTUs were manually filtered by excluding reads equal to or less than the negative control included in the analysis. The resulting CatL OTUs were used to construct a reference taxonomy database using the top hit result of BLASTn (BLASTN, RRID:SCR_001598) (49) above 90% identity and 100% query coverage. Finally, taxonomy was assigned to the CatL OTUs using “amptk taxonomy.” The generated CatL OTUs were assigned to each *Trypanosoma* spp., *T. congolense* subgroup (savannah, forest, kilifi) (13), and reported CatL clades based on the result of the top hit of the BLASTn homology search. *Trypanosoma vivax* CatL OTUs were assigned to their lineages (*T. vivax* and *T. vivax*-like) (21, 25–27) when they clustered with the reported sequences. All CatL OTU sequences generated in this study have been deposited to GenBank under Accession Numbers MT673751 to MT673783.

### Phylogenetic Analyses

Nucleotide sequences of each CatL OTU and reference sequences were aligned using MAFFT online v7 (MAFFT, RRID:SCR_011811) (50). The aligned sequences with 257 nucleotides were then used to construct neighbor-joining trees (51) using MEGA X (MEGA Software, RRID:SCR_000667) (52). The evolutionary distances were computed using the Maximum Composite Likelihood method (53) and default parameters with 10,000 bootstraps. The tree was visualized and annotated using iTOL v5.5 (iTOL, RRID:SCR_018174) (54).

### Statistical Analyses

Statistical analyses and visualization of other data were done in R v3.6.1 (R Project for Statistical Computing, RRID:SCR_001905) (55). For each pairwise comparisons of *Trypanosoma* spp. prevalence and PCV values, statistical significance (*p* < 0.05) was assessed using the Wilcoxon rank sum test with Bonferroni correction.

### Ethics Statement

This study was conducted under ethics approval Ref. No. 2019-Feb-081 (ERES Converge IRB, Lusaka, Zambia).

## Results

### *Trypanosoma* spp. Prevalence Based on Microscopy, ITS1 PCR, and SRA PCR

Results for microscopy, ITS1 PCR, SRA PCR, and CatL sequencing for all individual samples can be found in Supplementary Table 1. In summary, seven of 105 (6.67%) cattle thin blood smears were positive for *Trypanosoma* spp. by microscopy in Ntubya village. All animals from other villages were microscopically negative for *Trypanosoma* spp., and Ntubya had significantly higher prevalence of *Trypanosoma* spp. by microscopy compared to New ngoma and Basanga (Table 1 and Supplementary Table 2). In ITS1 PCR, 63 of 498 cattle (12.65%) were positive for one or more *Trypanosoma* spp. (Table 1). When compared between villages, Ntubya had the highest prevalence (32 of 105 cattle, 30.48%), followed by New Ngoma (12/102, 11.77%), Kaminza (8/85, 9.41%), Basanga (7/116, 6.03%), and Iyanda (4/90, 4.44%). Ntubya had significantly higher prevalence of the total *Trypanosoma* spp. by ITS1 PCR compared to every other villages (Table 1 and Supplementary Table 2). Overall, the most abundant species in cattle samples was *T. vivax* (50/498, 10.04%), followed by *T. congolense* (8/498, 1.61%), and *Trypanozoon* (7/498, 1.41%). *T. godfreyi* and *T. simiae* were not detected from the cattle samples. *Trypanosoma vivax* was detected in all five villages, and was the most abundant species in Ntubya, Kaminza, and New Ngoma (Table 1 and Supplementary Figure 1). Ntubya had significantly higher prevalence of *T. vivax* by ITS1 PCR compared to every other villages (Table 1 and Supplementary Table 2). *Trypanosoma congolense* was detected in four villages, but not in Kaminza. *Trypanozoon* was detected in four villages, but not in New Ngoma, and was the most abundant species in Iyanda and Basanga (Table 1 and Supplementary Figure 1). Two multiple infections of different *Trypanosoma* spp. were found among the positive samples in Ntubya (2/32, 6.25%). They involved *T. vivax*/*Trypanozoon* and *T. vivax*/*T. congolense* (Table 1).

**Table 1:** Microscopy, ITS1 PCR, and SRA PCR results for cattle blood samples in each village.

**Village**	**No. of samples**	**Microscopy**		**ITS1 PCR**												**SRA PCR**	
				***T. vivax***		***T. godfreyi***		***T. simiae***		***Trypanozoon***		***T. congolense***		**Total**			
		***n***	**% (95% CI)**	***n***	**% (95% CI)**	***n***	**% (95% CI)**	***n***	**% (95% CI)**	***n***	**% (95% CI)**	***n***	**% (95% CI)**	***n***	**% (95% CI)**	***n***	**% (95% CI)**
Ntubya	105	7	6.67* (2.72–13.25)	29	27.62* (19.34–37.2)	0	0.00 (0.00–3.45)	0	0.00 (0.00–3.45)	1	0.95 (0.02–5.19)	4	3.81 (1.05–9.47)	32	30.48* (21.87–40.22)	7	6.67 (2.72–13.25)
Kaminza	85	0	0.00 (0.00–4.25)	7	8.24 (3.38–16.23)	0	0.00 (0.00–4.25)	0	0.00 (0.00–4.25)	1	1.18 (0.03–6.38)	0	0.00 (0.00–4.25)	8	9.41 (4.15–17.71)	1	1.18 (0.03–6.38)
Iyanda	90	0	0.00 (0.00–4.02)	1	1.11 (0.03–6.04)	0	0.00 (0.00–4.02)	0	0.00 (0.00–4.02)	2	2.22 (0.27–7.80)	1	1.11 (0.03–6.04)	4	4.44 (1.22–10.99)	5	5.56 (1.83–12.49)
New ngoma	102	0	0.00 (0.00–3.55)	11	10.78* (5.51–18.48)	0	0.00 (0.00–3.55)	0	0.00 (0.00–3.55)	0	0.00 (0.00–3.55)	1	0.98 (0.02–5.34)	12	11.76 (6.23–19.65)	8	7.84 (3.45–14.87)
Basanga	116	0	0.00 (0.00–3.13)	2	1.72 (0.21–6.09)	0	0.00 (0.00–3.13)	0	0.00 (0.00–3.13)	3	2.59 (0.54–7.37)	2	1.72 (0.57–2.87)	7	6.03 (2.46–12.04)	6	5.17 (1.92–10.92)
Total	498	7	1.41 (0.57–2.87)	50	10.04 (7.54–13.02)	0	0.00 (0.00–0.74)	0	0.00 (0.00–0.74)	7	1.41 (0.57–2.87)	8	1.61 (0.70–3.14)	63	12.65 (9.86–15.89)	27	5.42 (3.60–7.79)

The number of cattle blood samples, number of samples positive for each Trypanosoma spp. based on microscopic observation, and gel electrophoresis results of ITS1 PCR and SRA PCR are shown in the table. The numbers are the total of individual samples positive with one (single infection) or more (multiple infection) Trypanosoma spp. The prevalence by each test was compared between each village by Wilcoxon rank sum-test. P-values were adjusted by Bonferroni correction. (*p < 0.05. P-values for all pairwise comparisons can be found in Supplementary Table 2).

In tsetse flies, 37 of 86 (43.02%) *G. m. centralis* were positive for one or more *Trypanosoma* spp. (Table 2). Of the 37 positive samples, seven were multiple infections of either *T. vivax*/*T. simiae, T. vivax*/*T. congolense, T. simiae*/*T. congolense*, or *T. vivax*/*T. simiae*/*T. congolense* (Table 2). Forty-three of 212 (20.28%) *G. pallidipes* were positive for one or more *Trypanosoma* spp. Of the 43 positive samples, five were multiple infections of either *T. vivax*/*T. congolense, T. godfreyi*/*T. simiae* and *Trypanozoon*/*T. congolense* (Table 2). *Trypanosoma vivax* was most abundant (52/298, 17.45%), followed by *T. congolense* (26/298, 8.73%), *T. simiae* (9/298, 3.02%), *Trypanozoon* (5/298, 1.68%), and *T. godfreyi* (1/298, 0.34%). *Glossina morsitans centralis* had significantly higher prevalence of the total *Trypanosoma* spp., *T. vivax*, and *T. simiae* by ITS1 PCR compared to *G. pallidipes* (Table 2 and Supplementary Table 3). The proportion of multiple infections in the positive samples was 18.92 and 11.63% for *G. m. centralis* and *G. pallidipes*, respectively (Table 2).

**Table 2:** ITS1 PCR for each tsetse fly species.

**Tsetse fly** ** species**	**No. of** ** samples**	**ITS1 PCR**											
		***T. vivax***		***T. godfreyi***		***T. simiae***		***Trypanozoon***		***T. congolense***		**Total**	
		***n***	**% (95% CI)**	***n***	**% (95% CI)**	***n***	**% (95% CI)**	***n***	**% (95% CI)**	***n***	**% (95% CI)**	***n***	**% (95% CI)**
Gmc	86	29	33.72* (23.88–44.72)	0	0.00 (0.00–4.20)	7	8.14* (3.34–16.05)	1	1.16 (0.03–6.31)	8	9.30 (4.10–17.51)	37	43.02* (32.39–54.15)
Gp	212	23	10.85 (7.00–15.83)	1	0.47 (0.01–2.6)	2	0.94 (0.11–3.37)	4	1.89 (0.52–4.76)	18	8.49 (5.11–13.09)	43	20.28 (15.09–26.33)
Total	298	52	17.45 (13.32–22.24)	1	0.34 (0.01–1.86)	9	3.02 (1.39–5.66)	5	1.68 (0.55–3.87)	26	8.72 (5.78–12.52)	80	26.85 (21.9–32.26)

The number of tsetse fly DNA samples, number of samples positive for each Trypanosoma spp. based on the gel electrophoresis result of ITS1 PCR, total of individual samples positive with one or more Trypanosoma spp., and the number of samples positive for one (single infection) and more than one (multiple infection) Trypanosoma spp. for each tsetse fly species (Gmc, Glossina morsitans centralis; Gp, G. pallidipes) is shown in the table. The prevalence by each test was compared between tsetse fly species by Wilcoxon rank sum-test. P-values were adjusted by Bonferroni correction. (*p < 0.05. P-values for all pairwise comparisons can be found in Supplementary Table 3).

Twenty-seven of 498 cattle (5.42%) were positive for SRA PCR (Table 1). In descending order, the prevalence in the villages was New Ngoma (8/102, 7.84%), Ntubya (7/105, 6.67%), Iyanda (5/90, 5.56%), Basanga (6/116, 5.17%), and Kaminza (1/85, 1.18%). Multiple false-positive bands were identified from the tsetse fly samples after SRA PCR. Since no negative controls with PCR grade water turned positive in any batch, contamination during PCR and PCR preparation had not occurred. Therefore, contamination of the SRA gene during DNA extraction was suggested, and therefore all tsetse fly samples were excluded from the SRA PCR experiments.

### The CatL OTU Diversity and Abundance Within Cattle and Tsetse Fly Samples

**Figure 1:**
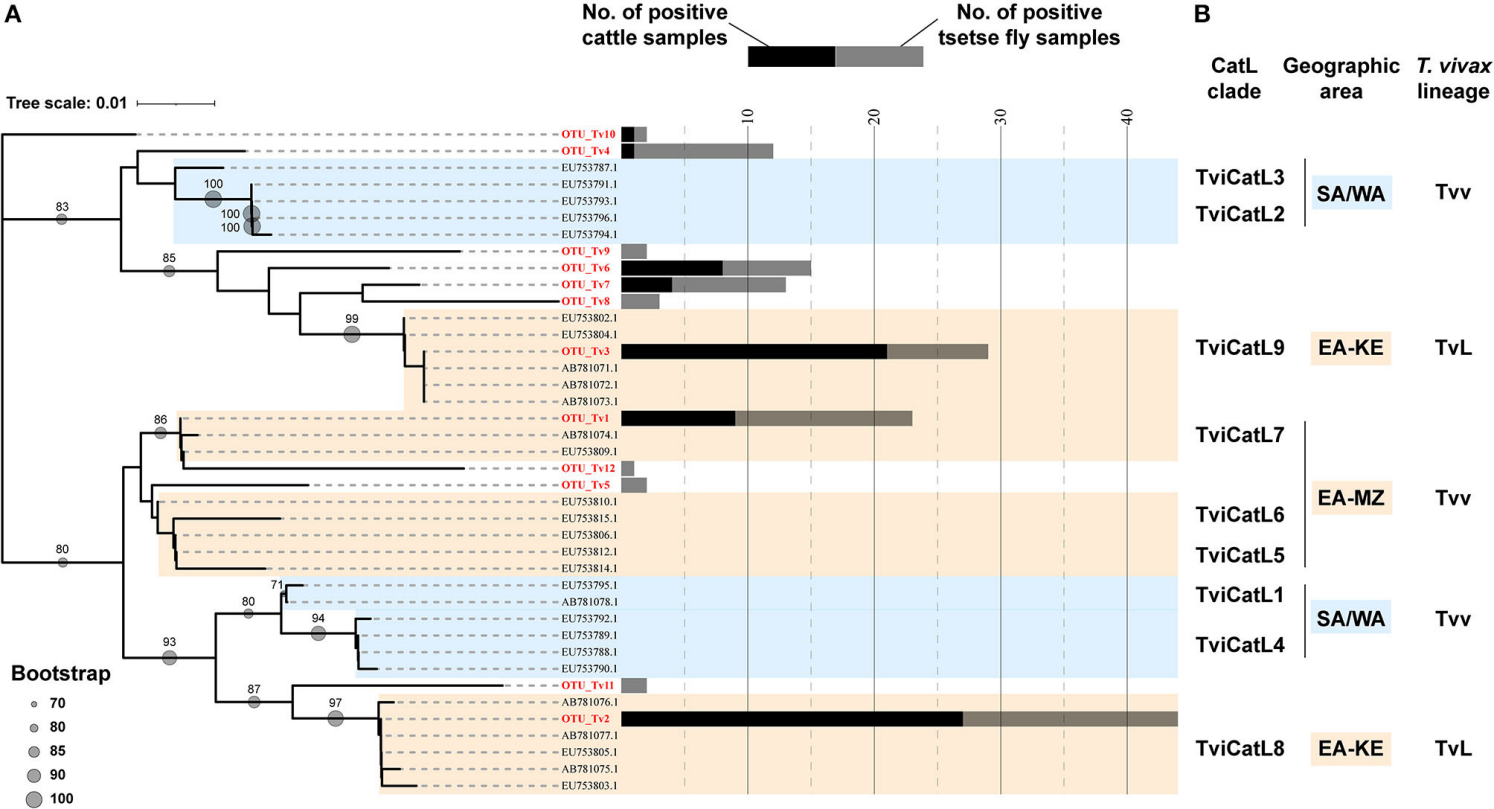
Neighbor-joining tree of 12 CatL OTUs and reference sequences for *Trypanosoma vivax*. **(A)** Phylogenetic relationship of the 12 CatL OTUs belonging to *T. vivax*. Bootstrap values above 70 are shown. The number of samples positive for each CatL OTUs are shown in bar graphs (black: number of cattle samples, gray: number of tsetse fly samples). **(B)** Nodes are defined with CatL clades (25) and *T. vivax* lineages (*T. vivax* and *T. vivax*-like) (27) reported for the corresponding reference sequences. SA/WA, isolates from South America and West Africa; EA-KE, isolates from Kenya and Zambia; EA-MZ, isolates from Mozambique and Zambia; Tvv, *T. vivax* lineage; TvL, *T. vivax*-like lineage.

**Figure 2:**
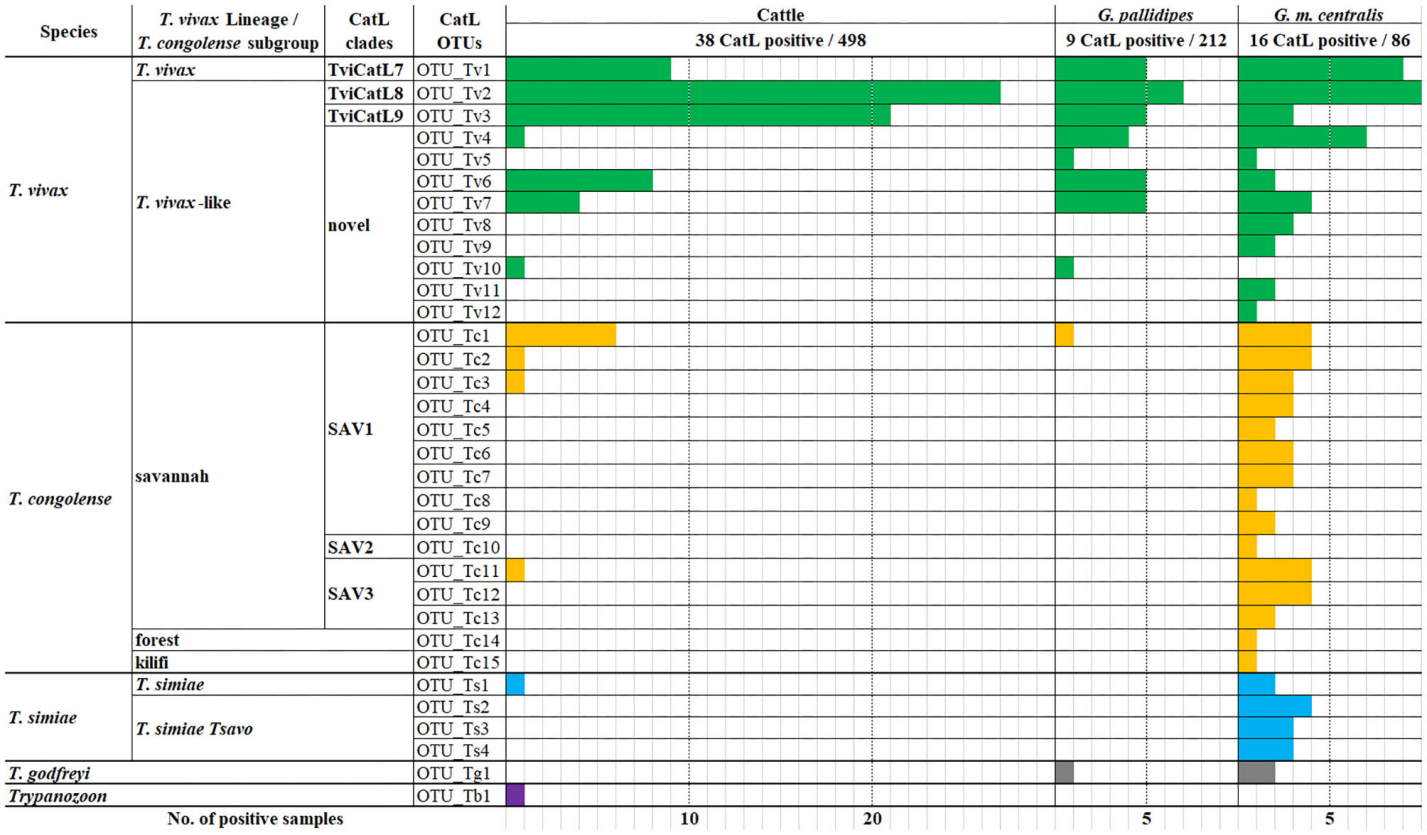
Number of samples positive for each CatL OTU. The number of samples positive for each CatL OTU is shown in the bar graph for cattle, *G. pallidipes*, and *G. m. centralis*.

A total of 143 cattle and tsetse fly samples that were positive for ITS1 PCR were subjected to CatL PCR and MiSeq amplicon sequencing. Among the ITS1 PCR-positive samples, 91 were positive for CatL PCR, and resulted in using 63 samples for downstream analysis after sequencing. The reduction was caused presumably by the difference in sensitivity between the ITS1 PCR and CatL PCR, and by the final reads filtering using the negative control. As a result, 33 CatL OTUs were generated and assigned to each *Trypanosoma* spp. (Supplementary Figure 3). Three *T. vivax* CatL OTUs clustered with reported CatL clades (Figure 1). On the other hand, nine CatL OTUs, OTU_Tv4–12, did not cluster with any of the reference sequences and were considered to be included in novel CatL clades (Figure 1). The phylogenetic relationship of the CatL OTUs and reference sequences of all *Trypanosoma* spp. are shown in Supplementary Figure 3. The number of CatL OTUs assigned to *T. vivax, T. congolense, T. simiae, T. godfreyi*, and *Trypanozoon* were 12, 15, four, one, and one, respectively (Figure 2). Among them, OTU_Tv1, 2, 3, 4, 6, 7, and 10 (*T. vivax*), OTU_Tc1, 2, 3, and 11 (*T. congolense*), and OTU_Ts1 (*T. simiae*) were found in cattle and tsetse fly samples. The results represent simultaneous infection of samples by *Trypanosoma* spp. with different CatL OTUs. The most abundant CatL OTU in both cattle and tsetse flies was OTU_Tv2. OTU_Tb1 (*Trypanozoon*) was only detected in cattle. CatL OTUs found only in tsetse flies included OTU_Tv5, 8, 9, 11, and 12 (*T. vivax*), OTU_Tc4, 5, 6, 7, 8, 9, 10, 12, 13, 14, and 15 (*T. congolense*), OTU_Ts2, 3, and 4 (*T. simiae*), and OTU_Tg1 (*T. godfreyi*) (Figure 2 and Supplementary Figures 4, 5). *Glossina morsitans centralis* had the highest diversity of CatL OTUs, in which all CatL OTUs, except OTU_Tv10 and OTU_Tb1, were detected (Figure 2 and Supplementary Figure 5).

### Mean PCV Comparisons Between Villages, ITS1 PCR Result, and CatL OTU Result

**Figure 3:**
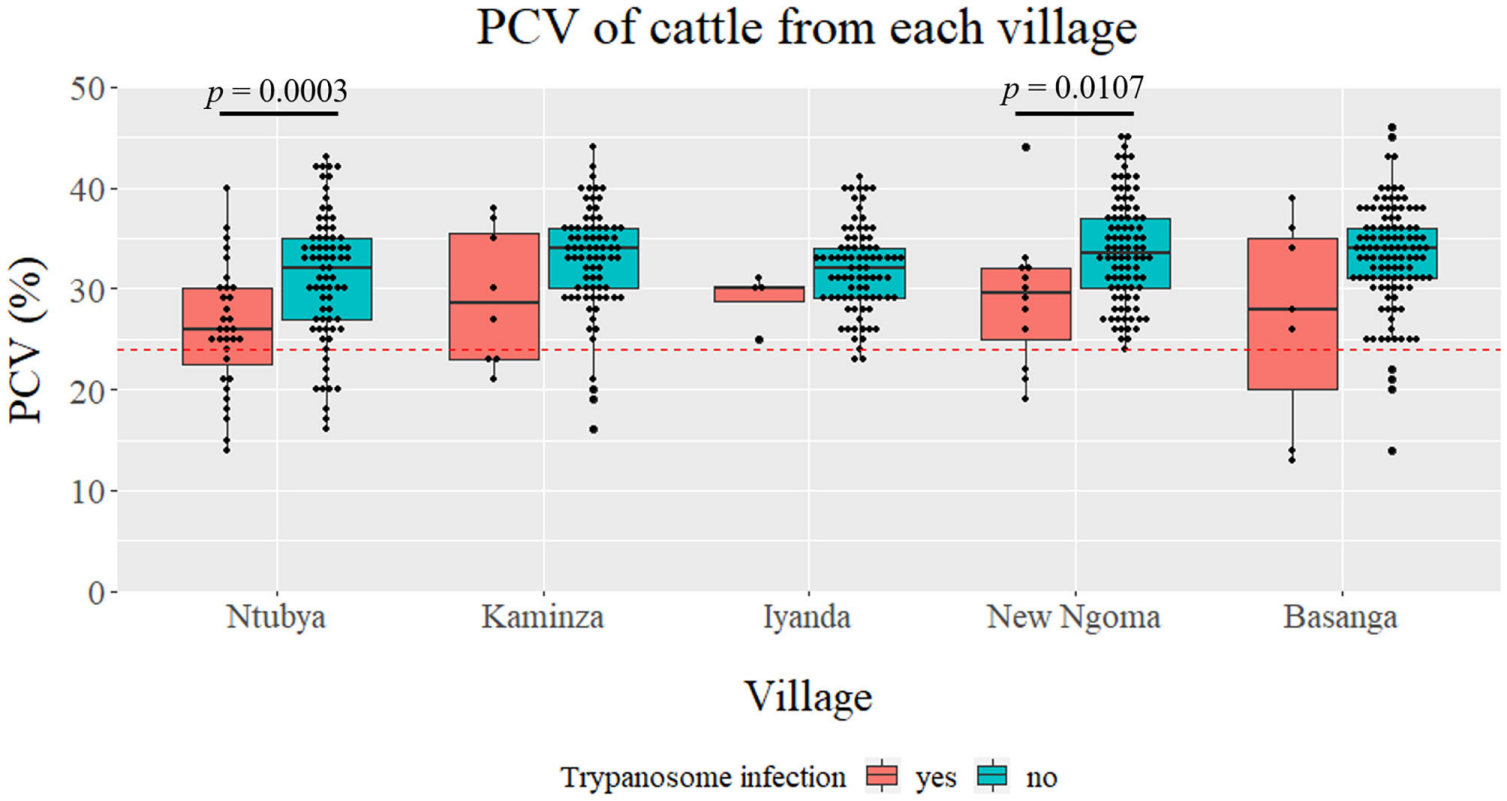
Comparison of packed cell volume (PCV) between villages. The PCV for ITS1 PCR positive (regardless of the species) and negative cattle are shown as boxplots for each village. Statistical significance was assessed by Wilcoxon rank sum-test (*p* < 0.05).

**Figure 4:**
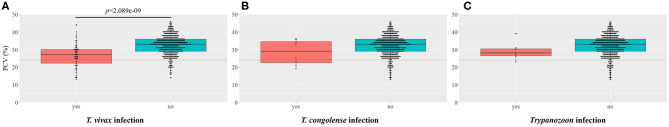
Comparison of packed cell volume (PCV) between *Trypanosoma* spp.-infected and non-infected cattle. The boxplot shows the comparison of the PCV between **(A)** *T. vivax*, **(B)** *T. congolense*, **(C)** *Trypanozoon*-positive and negative cattle samples (confirmed by ITS1 PCR). Statistical significance was assessed by Wilcoxon rank sum-test (*p* < 0.05).

The mean PCV value of cattle from each village, regardless of the *Trypanosoma* spp. infection status, was Ntubya 29.57 [standard deviation (SD): 6.80, 95% confidence interval (CI): 28.27–30.87], Kaminza 32.54 (SD: 5.40, 95% CI: 31.39–33.69), Iyanda 31.76 (SD: 4.31, 95% CI: 30.83–32.67), New Ngoma 33.18 (SD: 5.63, 95% CI: 32.08–34.27), and Basanga 32.75 (SD: 5.76, 95% CI: 31.70–33.80) (Supplementary Table 4). Ntubya had the lowest mean PCV, which was significantly lower than Kaminza, New ngoma, and Basanga (Supplementary Table 4). ITS1 PCR results grouped cattle samples into “infected” and “non-infected” groups. Infected cattle had significantly lower mean PCV in Ntubya and New Ngoma (Figure 3). When the cattle samples were grouped by the infection status according to each *Trypanosoma* spp., animals infected with *T. vivax* had significantly lower mean PCV compared with non-infected cattle (Figure 4A). Cattle infected with *T. congolense* and *Trypanozoon* also had a tendency of lower PCV compared to non-infected cattle, but no statistical significance was observed (Figures 4B,C). To check confounding effects, multiple regression analysis was conducted by including the village, sex, age, and the outcome of ITS1 PCR for each *Trypanosoma* spp. as coefficients. As a result, *T. vivax* infection significantly decreased PCV values even after excluding all other coefficients (*p* = 0.002). In addition, cattle with single infection of OTU_Tv2 (*T. vivax*-like, OTU pattern 2) tended to have lower mean PCV compared to negative cattle (OTU pattern 0) (Supplementary Figure 6).

## Discussion

In our study, cattle infected with *T. vivax* had significantly lower mean PCV than non-infected cattle, indicating that *T. vivax* is the major *Trypanosoma* spp. causing anemia in this area. Moreover, since only seven cattle had a detectable level of parasitemia by microscopy, it is assumed that *T. vivax* causes chronic bovine trypanosomosis with low parasitemia. This assumption agrees with the report of prolonged *T. vivax* infections with long aparasitemic intervals (56). Moreover, there was a tendency toward a lower mean PCV for cattle infected with *T. congolense* compared with non-infected cattle, but no significant difference was observed. In East and Southern Africa, *T. congolense* has been reported to be more pathogenic to cattle than *T. vivax* (56), and there has been a focus on *T. congolense* as the agent of AAT. Our results re-emphasize the importance of *T. vivax*, along with *T. congolense*, as a source of anemia. Monitoring *T. vivax* would be especially crucial since it can be mechanically transmitted and become widespread in regions not infested with tsetse flies. Furthermore, bovine trypanosomosis research and control programs in tsetse fly infested areas should also include biting flies other than tsetse flies.

Notably, we have detected a variety of CatL OTUs, with pronounced diversity in *T. vivax* (*T. vivax* and *T. vivax*-like) and *T. congolense* savannah. Some of the *T. vivax* CatL OTUs detected in this study were consistent with the CatL clades (TviCatL7, TviCatL8, TviCatL9) reported in other East and southern African countries (21, 25, 39). The findings were expected considering the geographical location of the study area. TviCatL7 contains isolates from Mozambique nyala and Zambian cattle, which were confirmed as *T. vivax* lineages that are closely related to those from West Africa and South America (21, 39). We detected one CatL OTU in this clade (OTU_Tv1), which was found in cattle and tsetse flies. TviCatL8 (OTU_Tv2) and TviCatL9 (OTU_Tv3) is included in *T. vivax*-like lineages, which to date have only been detected from tsetse fly infested areas in East Africa. Cattle with single infection of OTU_Tv2 (*T. vivax*-like, clade TviCatL8) tended to have lower PCV values than those that were negative for all OTUs, and cattle with single infection of OTU_Tv1 (*T. vivax*, clade TviCatL7) had a mean PCV value that was comparable with cattle that were negative for all OTUs. Therefore, parasites with different CatL OTUs may differ in their pathogenesis against cattle.

The rich CatL OTU diversity noted presently within *G. m. centralis* was remarkable. Except for OTU_Tv10 and OTU_Tb1, all OTUs were detected. Similar results were shown in Mozambique, where the highest diversity based on gGAPDH and ITS rDNA were identified in tsetse fly (*G. m. morsitans* and *G. pallidipes*) samples, and less diversity within cattle and wild animal samples (27). Together with our results, these findings support the possibility of a range of different variants emerging through recombination in tsetse flies, of which some adapted to cattle and spread across East Africa and West Africa (27). Different host susceptibility between different *Trypanosoma* spp. and *T. congolense* subgroups have been reported (57), which may be occurring within the *T. congolense* subgroups or *T. vivax* lineages. The difference in susceptibility may be caused by the host preference of the parasite or the immune system of the animal host. However, more stringent classification of *T. vivax* taxonomy and population genomic studies on isolates will be needed to further assess these possibilities of recombination within tsetse flies and adaptation to cattle. On the contrary, the CatL OTU diversity in *G. pallidipes* was low and comparable with what was observed in cattle. The difference in bloodmeal host preference, host abundance, distribution, and dispersal rate between the two tsetse fly species may have affected the difference in the CatL OTU diversity. Different bloodmeal preferences have been reported within the morsitans group of tsetse flies, where *G. pallidipes* was more likely to take a bloodmeal from bovids, such as bushbuck, buffalo, and eland, and *G. m. morsitans* preferred suids (warthog) over bovids (bushbuck) (58). The distribution of *G. pallidipes* in the Kafue area may be strictly restricted (43), and the dispersal rates may be small compared to *G. m. centralis* (59). Therefore, it is hypothesized that the *G. pallidipes* sampled in this study may have had limited access to the variety of wildlife, and preferentially took their bloodmeal from cattle, which resulted in the similar CatL OTU diversity observed between *G. pallidipes* and cattle. However, careful consideration of this hypothesis is needed, since the decreased OTU diversity observed in *G. pallidipes* may have been affected by bias associated with trapping methods. *G. pallidipes* samples were all trapped by epsilon traps, whereas a majority of *G. m. centralis* samples were trapped by the mobile traps. Furthermore, we did not include other hematophagous flies, such as Tabanids or *Stomoxys* spp., which are likely to be responsible for mechanical transmission of *T. vivax* between cattle. Clarifying the CatL OTU diversity in these biting flies would increase the understanding of the dynamics of *T. vivax* within the ecosystem.

The overall prevalence of *T. b. rhodesiense* confirmed by SRA PCR was 5.42%, with the highest value in New Ngoma (7.84%) and the lowest in Kaminza (1.18%) (Table 1). This prevalence is surprising since there has been no recent official report of HAT in any of the sampled villages. The prevalence in this study was relatively high compared to other *T. b. rhodesiense* prevalence studies conducted in Zambia (60). In an extreme case of the HAT outbreak in the Soroti district of Uganda caused by the importation of cattle from HAT endemic area, the prevalence of *T. b. rhodesiense* in cattle assessed by SRA PCR was estimated to be 18% (61). Although the prevalence level in our case was not as high, the Kafue ecosystem is considered as an old HAT focus with a re-emerging risk since the last case in 2016 (4). A study conducted in the same Kafue ecosystem detected *T. b. rhodesiense* in buffalos, a sable antelope, and a vervet monkey using SRA PCR, with respective prevalence values of 9.40% (5/53), 12.50% (1/8), and 100% (1/1) (62). The collective findings indicate that *T. b. rhodesiense* is circulating within free-ranging wildlife and human-owned cattle. This could be a concern to the community and requires careful monitoring.

In summary, combining CatL PCR with next generation sequencing is useful in illustrating *Trypanosoma* spp. diversity, especially for *T. vivax* and *T. vivax*-like trypanosomes. Further studies using isolates belonging to different CatL clades, such as population genomic studies to test recombination or *in vivo* pathogenesis tests, could clarify the epidemiology and relationship of these parasites with disease manifestation in cattle.

## Data Availability Statement

The datasets generated in this study can be found in online repositories. The names of the repository/repositories and accession number(s) can be found below: https://www.ncbi.nlm.nih.gov/genbank/, MT673751 to MT673783.

## Ethics Statement

The animal study was reviewed and approved by ERES Converge IRB, Lusaka, Zambia. Written informed consent was obtained from the owners for the participation of their animals in this study.

## Author Contributions

YN, KH, and JY: conceptualization, study design, and drafting of manuscript. YN, VD, YQ, MS, and BN: field sampling. YN: laboratory experiment. YN and RO: data analysis. YN, KH, VD, YQ, RO, MS, CS, BN, and JY: revising and final approval of the manuscript. All authors contributed to the article and approved the submitted version.

## Conflict of Interest

The authors declare that the research was conducted in the absence of any commercial or financial relationships that could be construed as a potential conflict of interest.
