# Supplementary material for: Genetic Diversity of African Trypanosomes in Tsetse Flies and Cattle From the Kafue Ecosystem
Source: Front Vet Sci. 2021 Jan 27;8:599815. doi: 10.3389/fvets.2021.599815 (PMC7873289; doi:10.3389/fvets.2021.599815)
Supplement: Supplementary file 1 [file Data_Sheet_1.docx]

Supplementary Material


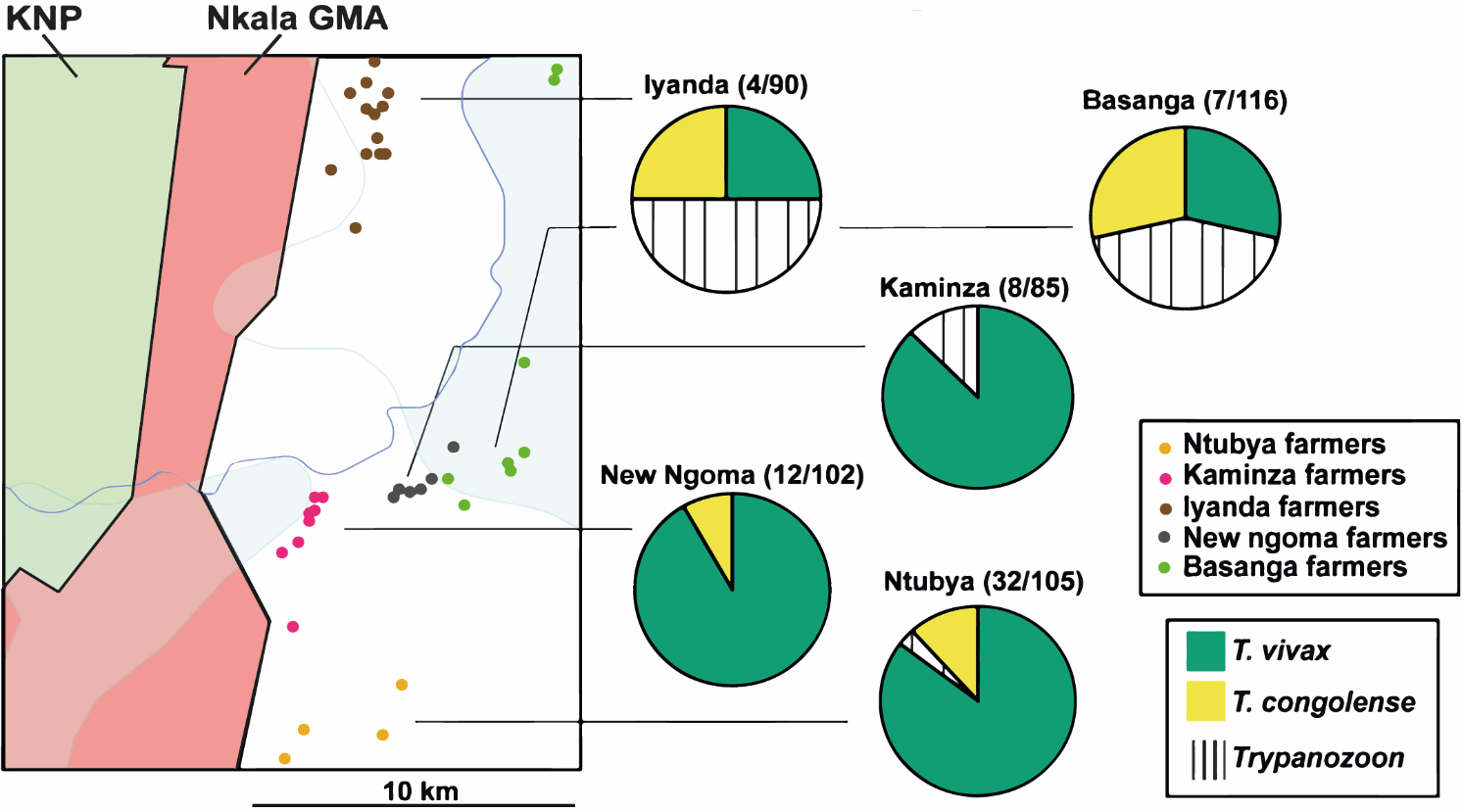


**Supplementary Figure 1. Geographic distribution of ITS1 PCR positive cattle samples.** The result of ITS1 PCR grouped per village is shown in the figure. The colored dots on the map represents each farm location where the sampling was taken place. Next to the name of the village shows the number of positive samples/total samples tested. The proportion of each *Trypanosoma* spp. detected within the group is shown as a pie graph, which size is adjusted to be the same across different number of samples.


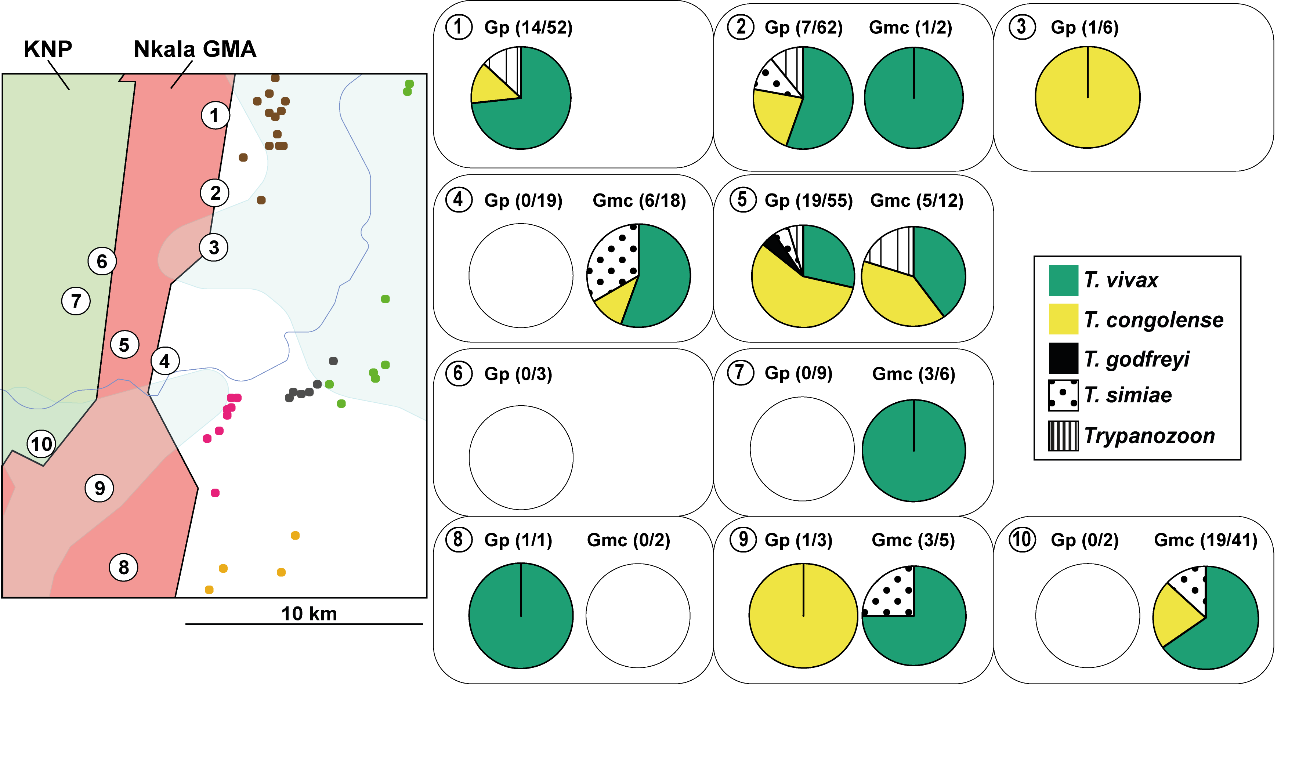


**Supplementary Figure 2. Geographic distribution of ITS1 PCR positive tsetse fly samples.** The result of ITS1 PCR for *Glossina pallidipes* (Gp) and *G. morsitans centralis* (Gmc), grouped per geographic area is shown in the figure. The encircled numbers on the map shows each location where the trapping was conducted. The number of positive samples/total samples tested are shown in parenthesis. The proportion of each *Trypanosoma* spp. detected within the group is shown as a pie graph, which size is adjusted to be the same across different number of samples.


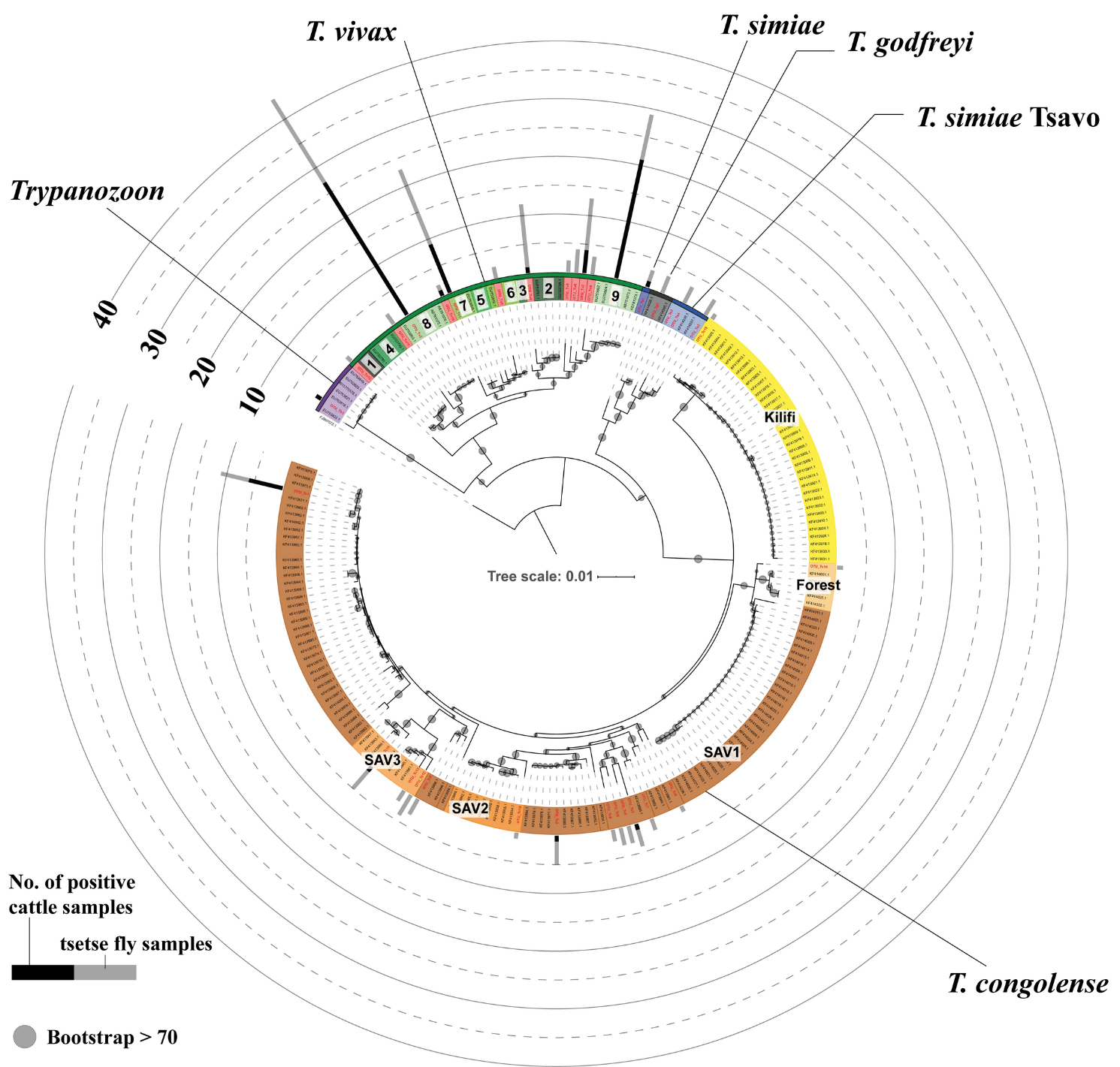


**Supplementary Figure 3. Neighbor-Joining tree of 33 OTUs and CatL reference sequences.** Total of 213 sequences with 33 OTUs detected in this study and 180 reference sequences were analyzed. There was a total of 257 positions in the final dataset. Bootstrap values more than 70 are shown in proportionate size circles for each node. The outer layer defines the *Trypanosoma* species (*Trypanozoon*, *T. vivax*, *T. simiae*, *T. simiae* Tsavo, *T. godfreyi*, and *T. congolense*), and the inner layer describes the CatL clades according to the reference sequences (*T. vivax*: TviCatL1, TviCatL2, TviCatL3, TviCatL4, TviCatL5, TviCatL6, TviCatL7, TviCatL8, TviCatL9; *T. congolense*: SAV1, SAV2, SAV3, Forest, Kilifi). The number of samples positive for each OTUs are shown as bar graphs (black: number of cattle samples, grey: number of tsetse fly samples).


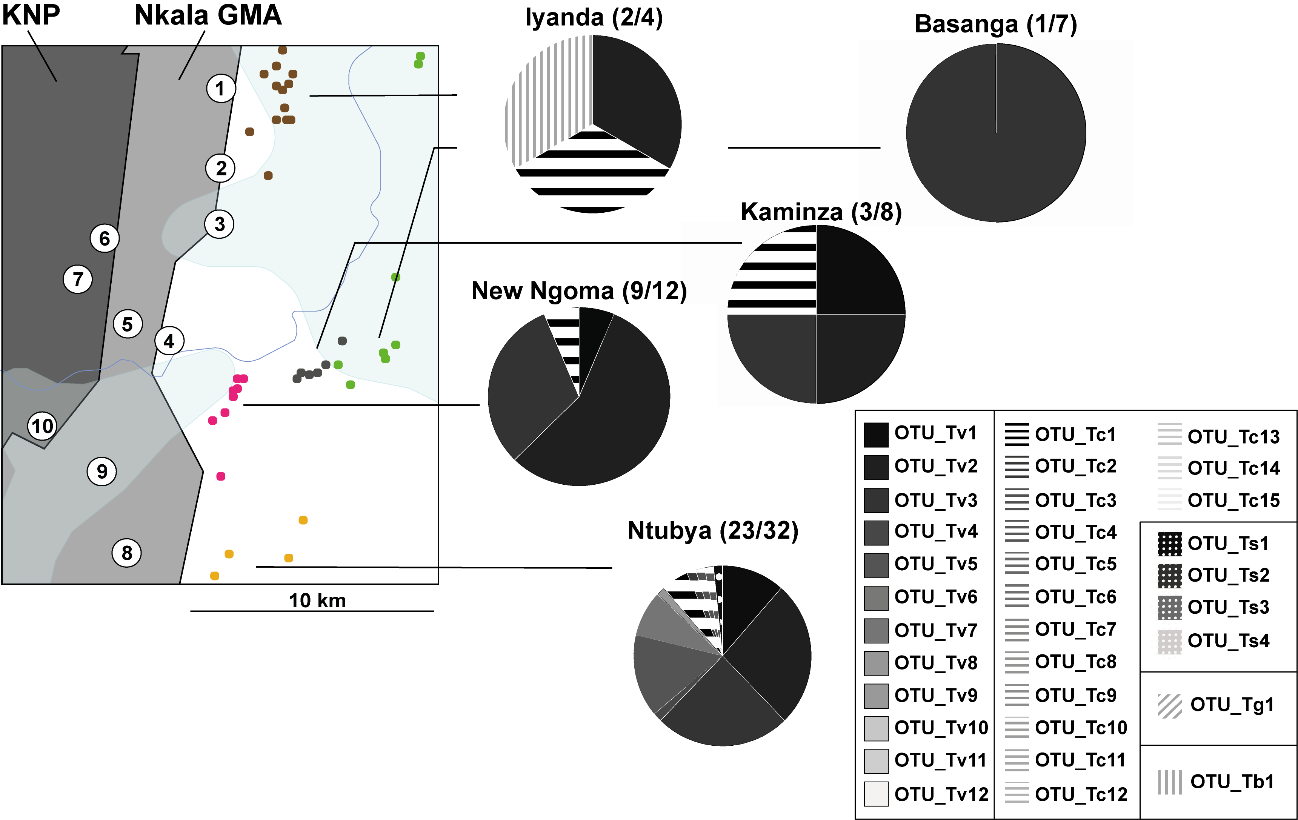


**Supplementary Figure 4. Distribution of CatL OTUs detected in cattle samples.** The number of OTUs detected as a result of CatL PCR and sequencing, grouped per village is shown in the figure. The colored dots on the map represents each farm location where the sampling was taken place. Next to the name of the village shows the number of positive samples/total samples tested. The proportion of each OTUs detected within the group is shown as a pie graph, which size is adjusted to be the same across different number of samples.


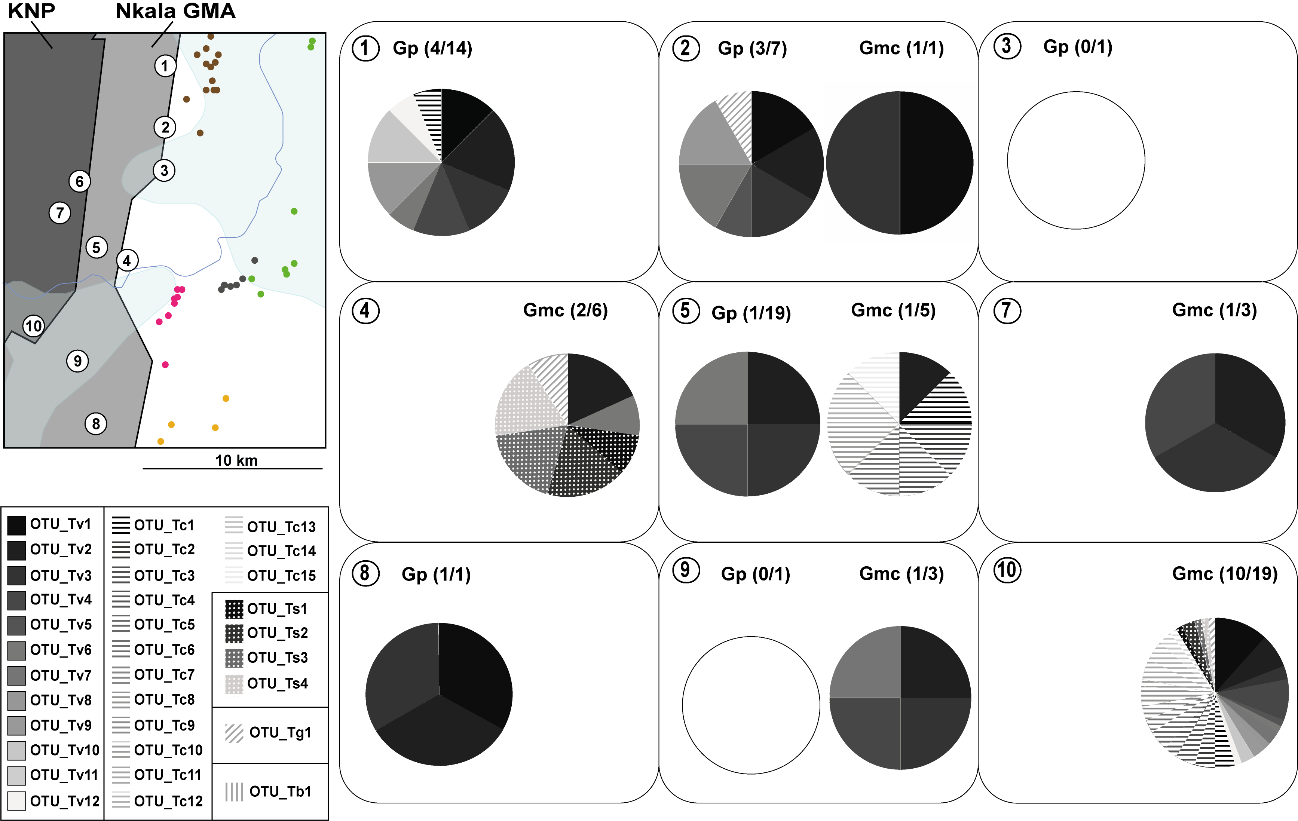


**Supplementary Figure 5. Distribution of CatL OTUs detected in tsetse fly samples.** The number of OTUs detected as a result of CatL PCR and sequencing, grouped per geographic area is shown in the figure. The encircled numbers on the map shows each location were the trapping was conducted. The number of positive samples/total samples tested are shown in parenthesis. The proportion of each OTUs detected within the group is shown as a pie graph, which size is adjusted to be the same across different number of samples. Gp: *Glossina pallidipes*, Gmc: *G. morsitans centralis*.


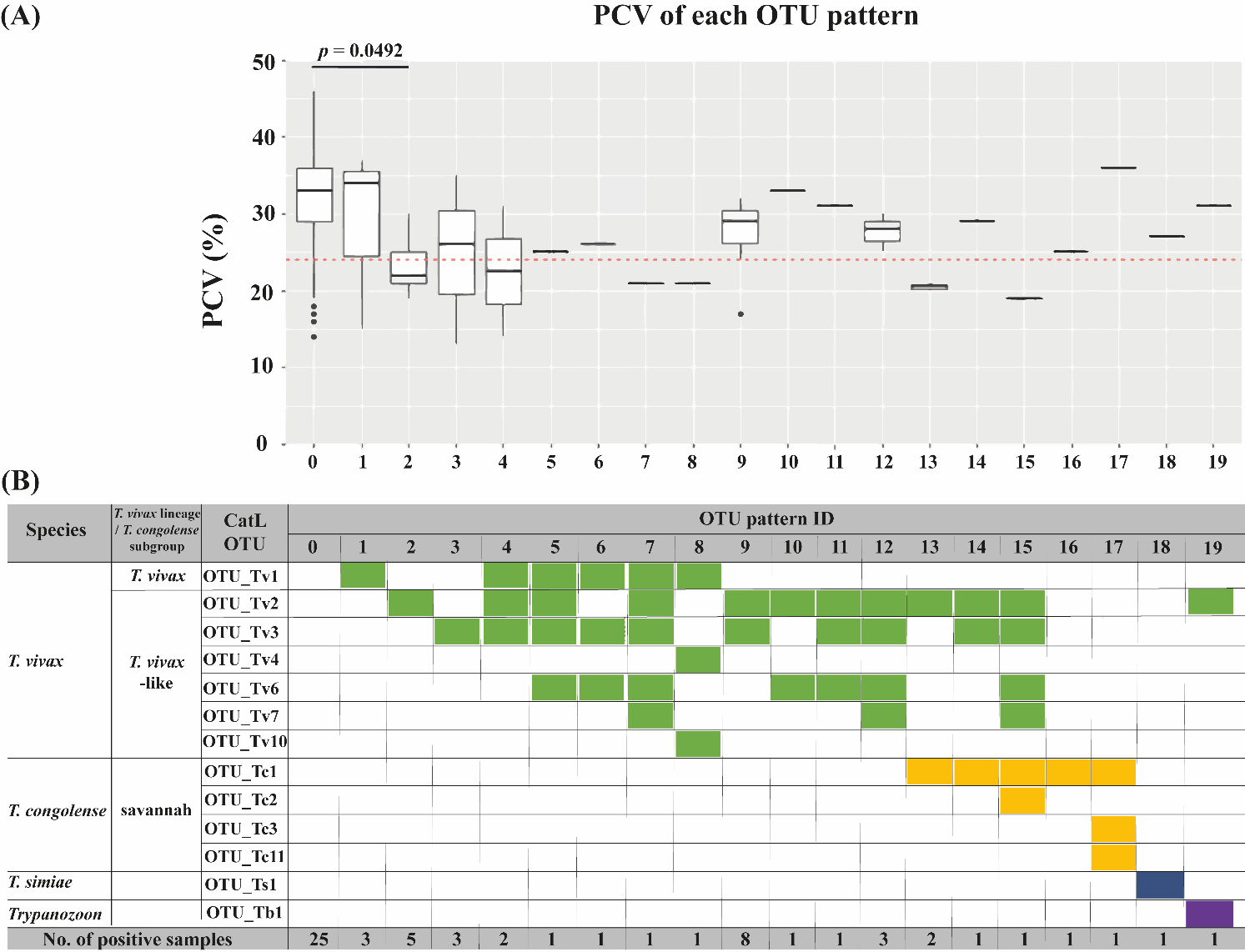


**Supplementary Figure 6. Comparison of packed cell volume between groups of cattle with different OTU patterns.** Each cattle were assigned to a group according to a pattern of which OTU they were positive for (confirmed by CatL PCR and sequencing). (A) Comparison of the packed cell volume (PCV) between different OTU patterns. (B) The table shows which pattern is positive to which OTU(s). OTU pattern 0 indicates samples which were negative for all OTUs. Statistical significance was assessed by Wilcoxon rank sum test (*p* < 0.05). However, all comparisons have resulted to be insignificant after P-value adjustment by Bonferroni correction.
